# Imagable 4T1 model for the study of late stage breast cancer

**DOI:** 10.1186/1471-2407-8-228

**Published:** 2008-08-09

**Authors:** Kai Tao, Min Fang, Joseph Alroy, G Gary Sahagian

**Affiliations:** 1 Department of Physiology, Tufts University School of Medicine, Boston, Massachusetts, 02111, USA; 2 Departments of Pathology, Tufts University School Cummings Veterinary Medicine and Tufts-New England Medical Center, Boston, Massachusetts, 02111, USA

## Abstract

**Background:**

The 4T1 mouse mammary tumor cell line is one of only a few breast cancer models with the capacity to metastasize efficiently to sites affected in human breast cancer. Here we describe two 4T1 cell lines modified to facilitate analysis of tumor growth and metastasis and evaluation of gene function *in vivo*. New information regarding the involvement of innate and acquired immunity in metastasis and other characteristics of the model relevant to its use in the study of late stage breast cancer are reported.

**Methods:**

The lines were engineered for stable expression of firefly luciferase to allow tracking and quantitation of the cells *in vivo*. Biophotonic imaging was used to characterize growth and metastasis of the lines *in vivo *and an improved gene expression approach was used to characterize the basis for the metastatic phenotype that was observed.

**Results:**

Growth of cells at the primary site was biphasic with metastasis detected during the second growth phase 5–6 weeks after introduction of the cells. Regression of growth, which occurred in weeks 3–4, was associated with extensive necrosis and infiltration of leukocytes. Biphasic tumor growth did not occur in BALB/c SCID mice indicating involvement of an acquired immune response in the effect. Hematopoiesis in spleen and liver and elevated levels of circulating leukocytes were observed at week 2 and increased progressively until death at week 6–8. Gene expression analysis revealed an association of several secreted factors including colony stimulatory factors, cytokines and chemokines, acute phase proteins, angiogenesis factors and ECM modifying proteins with the 4T1 metastatic phenotype. Signaling pathways likely to be responsible for production of these factors were also identified.

**Conclusion:**

The production of factors that stimulate angiogenesis and ECM modification and induce hematopoiesis, recruitment and activation of leukocytes suggest that 4T1 tumor cells play a more direct role than previously appreciated in orchestrating changes in the tumor environment conducive to tumor cell dissemination and metastasis. The new cell lines will greatly facilitate the study of late stage breast and preclinical assessment of cancer drugs and other therapeutics particularly those targeting immune system effects on tumor metastasis.

## Background

While investigation of the molecular basis of tumor metastasis has in large part focused on proliferation and dissemination of tumor cells from the primary tumor, later events that occur at sites of metastasis are most often responsible for patient mortality and morbidity. From a clinical standpoint, an understanding of the disease at metastatic sites is paramount since the number of breast cancer patients with detectable or occult metastases at the time of diagnosis is substantial and most patients will develop metastatic lesions at some point during the course of the disease. Metastasis is generally treated as a systemic disease with chemotherapy and/or radiation even though factors involved in establishment and growth of metastatic lesions differ from one site to the next and may differ in response to therapeutics. While currently used therapeutic regimens are capable of slowing the progression of metastatic disease, rarely is it possible to stop or reverse the process. Treatments that address the nature of metastatic disease at the site of metastasis could provide more effective therapeutic results for patients afflicted with the later stages of the disease.

A major impediment for the study of metastasis has been the availability of suitable models that faithfully represent the metastatic process as it occurs *in vivo*. Xenograft models in which human tumor cells are introduced into immunocompromised mice have been used extensively for the study of tumor growth and metastasis and to validate specific gene products as drug targets for cancer therapy. While some human xenograft models can approximate primary tumor growth in mice, replication of tumor metastasis is more problematic [1-3]. Human tumor cells generally metastasize poorly in mice and when metastasis does occur, unexpected metastatic characteristics are often observed. In contrast, murine tumor cell models often metastasize more effectively and display metastatic characteristics more similar to those observed in cancer patients [4]. Given the importance of microenvironment and tumor-host interactions in tumor cell behavior, this is not surprising. Syngeneic mouse models such as the 4T1 model described here also have the important advantage of allowing analyses to be carried out in animals with normal immune function. Because the immune system plays an important role in the development and progression of cancer, models that can be used in immunocompetent mice are essential for analysis of cancer progression and evaluation of therapeutics for cancer treatment.

The 4T1 mammary carcinoma cell line was originally isolated by Fred Miller and coworkers at the Karmanos Cancer Institute [5,6]. Its use has increased in recent years because of its high propensity to metastasize to bone and other sites [7,8]. When introduced orthotopically, 4T1 is capable of metastasis to several organs affected in breast cancer including lungs, liver and brain, as well as bone [7,9-11]. 4T1 sibling cell lines with different metastatic properties have been isolated and characterized. These lines were isolated from the same spontaneous arising BALB/c mammary tumor [5,6] but appear to have followed divergent pathways for acquisition of their metastatic phenotypes [12].

We have modified the 4T1 cell line for optimal use as a model for the study of late stage breast cancer. A modified line (4T1-12B) expressing high levels of firefly luciferase to allow non-invasive longitudinal imaging of *in vivo *growth and metastasis was isolated. A similar line (4T1-1V) was further modified by insertion of a FLP recombinase target (FRT) site into the 4T1 genome. The FRT site facilitates rapid generation of genetically modified isogenic cell lines for investigation of effector gene function. The extent and kinetics of metastasis to organs affected in human breast cancer indicated extensive colonization of lungs and liver in most animals within a six week period with lower efficiency of metastasis to bone, brain and other sites. Innate and adaptive immune responses were shown to play important roles in growth and metastasis of the lines in BALB/c mice. Analysis of gene expression comparing 4T1 and two of its non-metastatic sibling cell lines suggested prominent roles for several signaling pathways and secreted factors in directing microenvironmental changes within the tumor leading to tumor cell dissemination and metastasis.

## Methods

### Materials

The luciferase-containing pGL3-Control vector was obtained from Promega. The pKO-puro vector was from Stratagene. The pSHAG-1 vector was provided by Dr. G. Hannon at Cold Spring Harbor Laboratory. Other vectors including pcDNA5/FRT, pOG44, pFRT/lacZeo, and the Gateway Vector Conversion System Reading Frame Cassette C.1 were obtained from Invitrogen. Dulbecco's modified Eagle's medium (DMEM), Dulbecco's phosphate-buffered saline without calcium and magnesium (PBS), fetal bovine serum (FBS), newborn calf serum (NCS), non-essential amino acids (NEAA), penicillin, streptomycin and lipofectamine PLUS reagent were from Invitrogen. Puromycin and hygromycin were from Sigma. Luciferin was obtained from Caliper Life Sciences.

### Cell culture

The 67NR, 168FARN and 4T1 mouse mammary tumor cell lines were obtained from Dr. Fred Miller at Karmanos Cancer Institute. Cells were cultured in high glucose DMEM supplemented 5% FBS, 5% NCS, NEAA and antibiotics (100 units/ml penicillin and 100 μg/ml streptomycin) at 37°C in a humidified atmosphere containing 5% CO_2_. Except where indicated, analyses were performed on same passage cells within 2 weeks after thawing. All cell lines used in the study were tested and shown to be free of mycoplasma and viral contamination.

### Expression of luciferase and puromycin resistance in 4T1 cell lines

4T1 cells were cotransfected with firefly luciferase-containing pGL-3-Control vector and the puromycin resistance vector, pKO-puro, at a ratio of 10:1 using Lipofectamine PLUS as described by the vendor [Invitrogen]. Transfected cells were selected with puromycin at a final concentration of 10 μg/ml and several colonies were picked and expanded for analysis. Colonies displaying the highest level of luciferase expression were injected into mammary fat pads of female BALB/c mice and imaged 6 weeks later before and after sacrifice and necropsy, as described below. One cell line, designated 4T1-12B, which retained high level expression of luciferase in the absence of puromycin and displayed metastatic properties similar to the parental line, was retained for further analysis and modification. Sublines were obtained from the 4T1-12B line by limiting dilution cloning.

### Incorporation of FRT site into 4T1 cell line

To introduce the FLP recombinase target (FRT) site in the 4T1 genome, cells were transfected with pFRT/lacZeo using lipofectamine PLUS and selected with 100 μg/ml zeocin. Colonies were picked and expanded and six clonal lines with a single integration of the vector, as determined by Southern blotting, were identified. The expanded lines were analyzed for efficiency of transfection and targeting and for *in vivo *tumor growth and metastasis. One line, designated 4T1-1V, displayed growth and metastatic characteristics similar to the parental 4T1 line and efficient transfection and targeting to the FRT site with pcDNA5/FRT (Invitrogen) and was retained for further analysis.

### Construction of shRNA targeting vector

The pcDNA5/FRT targeting vector was modified to allow transfer of the shRNA expression cassettes from pSHAG-type shRNA expression vectors [13] to the pcDNA5/FRT vector by Gateway site-specific recombination. Resulting vectors can then be used to target shRNA expression cassettes to FRT sites in 4T1-1V and other FRT-containing cell lines for creation of isogenic cell lines. A Gateway cloning site was inserted into the vector by blunt end ligation of Reading Frame Cassette C.1 (Invitrogen) into the vector's Bgl II site.

### Knockdown of luciferase with shRNA targeting vector

To test the efficacy of the construct, an empty expression cassette and a cassette encoding a previously tested luciferase shRNA [13] were transferred to the modified pcDNA5/FRT from corresponding pSHAG-1 vectors. The 4T1-1V cells were then cotransfected with each construct and pOG44 vector at a ratio of 1:10 using Lipofectamine PLUS. Transfected cells were selected with hygromycin B at a final concentration of 200 μg/ml. The pOG44 vector encodes FLP recombinase which directs insertion of the modified pcDNA5/FRT targeting vector into the cell's FRT site; hygromycin B resistance is conferred upon insertion of the vector into the site.

Expanded clones of hygromycin B resistant cells as well as resistant cell pools from each transfection were then assayed for luciferase activity using a Turner Designs Model TD-20/20 Luminometer.

### Biophotonic imaging of animals and organs

Luciferase-expressing cell lines were plated at 40% confluency and cultured for 24 h. The cells were then trypsinized, washed, and resuspended in DMEM at 10^7 ^cells/ml and kept on ice before injection. Aliquots (100 μl) of the cells were injected into the no. 4 or no. 9 fatpad of 4–6 week old female BALB/c, athymic BALB/c nude or BALB/c SCID mice using a 26-gauge needle. Only cell preparations with viability > 97%, as determined by Trypan Blue exclusion, were used for injection.

At various times up to 6 weeks, animals were injected intraperitoneally with 100 μl of D-luciferin (10 mg/ml) in PBS, and after 10 min, imaged under anesthesia with 2.5% isofluorane in a Xenogen IVIS 200 biophotonic imager. At experimental endpoints, luciferin-injected animals were sacrificed and organs and hind limbs were removed and imaged within 15 minutes after injection. Luminescence is expressed as photons/sec/ROI (region of interest) minus background luminescence for a similarly sized region. All experiments with animals were carried out according to guidelines for the care and use of experimental animals and were approved by the Tufts University Institutional Animal Care and Use Committee.

### Chip hybridizations and analysis of expression data

Biotin-labeled cRNAs were prepared from 250 ng of total RNA using Ambion's TotalPrep RNA Amplification Kit. Chip hybridizations, washing, Cy3-streptavidin (Amersham Biosciences) labeling, and scanning were performed on an Illumina BeadStation 500 platform using reagents and protocols provided by the manufacturer. cRNA samples were hybridized to Illumina MouseRef-8 BeadChips which cover 24,048 RefSeq transcripts. The manufacturing principle of randomly distributing large populations of oligonucleotide-coated beads across the available positions on the chip enables 30 intensity measurements per feature on average, and produces quantitative results closely matching those obtained by Q-PCR [14].

Biotin-labeled cRNAs were prepared from 3 biological replicates of cultured 4T1, 67NR and 168FARN cells for hybridization to the chips. Cells were plated at 5 × 10^5 ^cells in 10 cm culture dishes and after 3 days, when the cells had reached confluence, the medium was changed and the cells were cultured for an additional 24 hours. Total RNA was isolated using the Absolutely RNA Kit from Stratagene and checked for integrity using an Agilent Lab-on-a-Chip Bioanalyzer.

Initial analysis of the data was carried out using Illumina's BeadStudio software. Raw data for each sample were background-subtracted and normalized using the "cubic spline" algorithm. Further statistical analysis of the data was carried out using programs associated with BRB Array Tools [15]. Differentially expressed genes for 4T1 samples relative to 67NR and 168FARN samples were determined separately with the Class Comparison program using the random variance model and a p value of 0.0001. Signals less than 10 were set to 10 to eliminate the inaccuracy of analyzing genes expressed at near background levels from being scored as differentially expressed. Genes that differed significantly (p < 0.0001) by > 2-fold for both comparisons (4T1/67NR and 4T1/168FARN) were considered those associated with the metastatic phenotype of the 4T1 cell line. Because of the high level of reproducibility that was achieved, a relatively high level of stringency (p < 0.0001) was chosen for selection of significant differences. As described in RESULTS, this permitted a very low level of false positives with minimal loss of true positives.

### Histochemistry and hematological analysis

Standard H & E staining of paraffin embedded tissue was used for histological examination of primary tumors and metastases. Stained sections were examined and photographed using an Olympus Vanox-T microscope and an Olympus U-PMTVC CCD camera. Blood from control and tumor bearing animals was collected by cardiac puncture and analyzed by the Pathology Department at Tufts University Cummings School of Veterinary Medicine using standard hematological procedures.

## Results

### Characteristics of luciferase-expressing 4T1 cell lines

The luciferase-expressing 4T1-12B cell line was cloned from 4T1 cells co-transfected with vectors encoding firefly luciferase and puromycin drug resistance. The level of luciferase expression is high enough to allow imaging of as few as 10 cells *in vitro *using a Xenogen IVIS 200 biophotonic imager; the cells are fully resistant to inclusion of 10 μg/ml of puromycin in the culture medium. The modified cell line displayed a doubling time of 12 hours in culture and a plating efficiency of 34%. Expression of luciferase persisted when the cells were cultured for extended periods (> 2 months) in the absence of selective pressure, a characteristic critical for reliable quantitation of tumor growth and metastasis *in vivo*.

**Figure 1:**
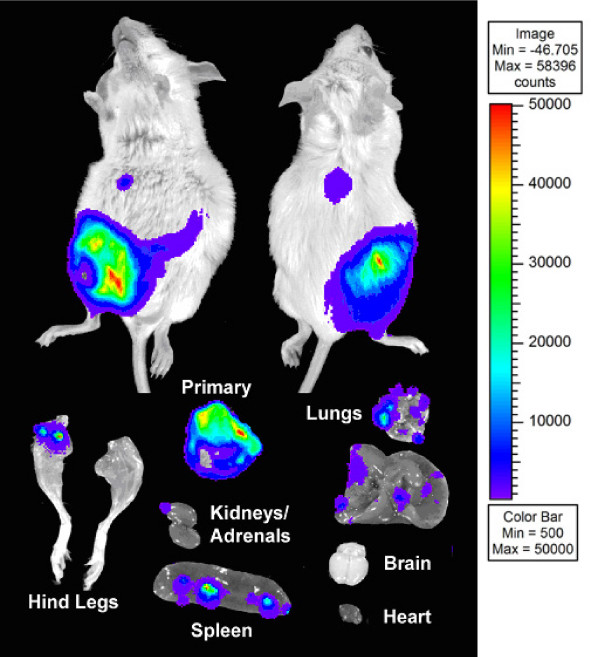
**Imaging of animals and organs at six weeks**. 4T1-12B cells (10^6^) were implanted into the mammary fat pad of a normal female BALB/c mouse. After six weeks the whole animal and organs were imaged as described in MATERIALS AND METHODS. The relationship between color and light intensity in arbitrary units (counts) for the whole animal images is given by the color bar at the right side of the figure.

Imaging of a representative female BALB/c mouse six weeks after mammary fat pad injection of 4T1-12B cells is shown in Figure 1. At six weeks, primary tumors generally reach a size of 1 cm^3 ^or more and metastasis to the thoracic region is apparent in most animals. Imaging of visceral organs and hind limbs revealed metastases in several organs including lungs, liver, bone and brain, sites affected in human breast cancer.

A compilation of results for several animals injected with the 4T1-12B line and several clones isolated from the modified line after extended time in culture is shown in Table 1. All animals injected with the 4T1-12B line displayed metastases in lungs at six weeks with substantial numbers displaying metastasis to liver (5/6), spleen (3/6) and bone (2/6). Metastases were occasionally found in lymph nodes, brain, intestine, kidneys and adrenals. Recloned sublines isolated from the 4T1-12B line displayed a similar spectrum of organ metastasis indicating that most if not all of the cells in the preparation are tumorigenic and metastatic and that the tumorigenic and metastatic properties of the cells are stable when cells are expanded for as many as 20 to 30 generations in culture.

**Table 1:** Summary of sites of metastasis for 4T1-12B^1^

**Cell Line**	**Primary**	**Lungs**	**Spleen**	**Liver**	**Bone**	**Other**
4T1-12B line	6(6)^2^	6(6)	3(6)	5(6)	2(6)	Brain 1(6) Intestine 1(6) Kidney 1(6)
4T1-12B recloned lines (5)	10(10)	8(10)	2(10)	3(10)	6(10)	Intestine 3(10) Kidney 1(10)

^1^Cells were introduced into fatpads of normal female BALB/c mice and imaged after 5–6 weeks as described in MATERIALS AND METHODS.

^2^Number of animals positive (total number of animals)

**Figure 2:**
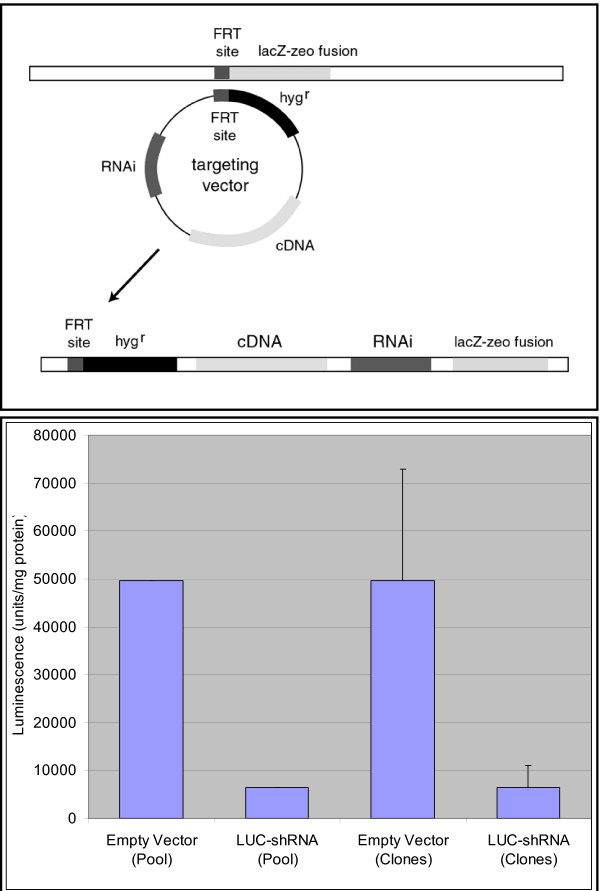
**Targeting shRNAs and cDNAs to FRT site in 4T1-1V**. **(Top) **The diagram shows the results of FLP recombinase-dependent insertion of the FRT targeting vector carrying a cDNA and/or siRNA, into the genome. The promoter driving expression of the lacZ-zeo fusion protein before insertion drives expression of the hygromycin resistance gene after insertion allowing hygromycin selection of cells that had undergone targeted insertion of the vector. **(Bottom) **Cells were cotransfected with the indicated vector and an expression vector encoding FLP recombinase. Cell pools and clones were isolated from the transfected cells and assayed for luciferase expression as described in MATERIALS AND METHODS. Light emission in arbitrary units per milligram of cell protein is shown for pools and clones transfected with empty targeting vector or targeting vector encoding luciferase shRNA. Error bars represent standard deviations for empty vector (n = 6) and luciferase siRNA vector (n = 7) transfected clones.

Generation of the 4T1-1V cell line involved transfection of the 4T1 line with luciferase and puromycin vectors and a vector containing a FLP recombinase targeting (FRT) site contained within a lacZ-zeo fusion protein expression cassette as described in MATERIALS AND METHODS. The 4T1-1V line was shown to contain a single site of integration of the FRT vector, to be readily susceptible to integration by FRT-containing targeting vectors, to stably express luciferase, and to have metastatic characteristics similar to the 4T1-12B line and its sublines. A plasmid containing a Gateway cloning site for insertion of small hairpin siRNA sequences was constructed from the FRT targeting vector, pcDNA5/FRT, and tested for its ability to be incorporated into the genome of the 4T1-1V line in an FRT-dependent manner (Figure 2). A vector carrying a previously tested sequence [13] encoding a small hairpin RNA for firefly luciferase was shown to effectively inhibit expression of luciferase in the 4T1-1V line.

### Progression of tumor growth and metastasis *in vivo*

**Figure 3:**
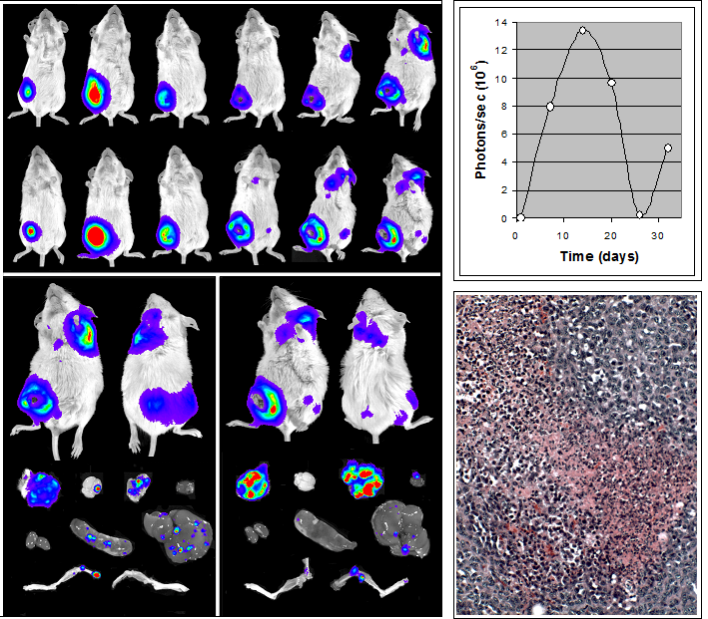
**Progression of tumor growth and metastasis**. **(Left) **4T1-1V cells (10^6^) were introduced into mammary fat pads of normal female BALB/c mice and the animals were imaged on a weekly basis for six weeks. The animals were sacrificed at the end of the sixth week and organs and hind limbs were removed and imaged. Images for two representative animals are shown. (**Top Right**) Quantitation of light emission from primary tumor over the six week period. (**Bottom Right**) H&E staining of a section from a primary tumor illustrating a central area of necrosis infiltrated by leukocytes and neoplastic cells at the periphery. The neoplastic cells are poorly differentiated and characterized by the presence of large hyperchromatic nuclei and relatively small amount of cytoplasm. Identifiable neutrophils and mast cells that appear to be located extravascularly were observed in non-necrotic areas of the tissue.

The results of a longitudinal study of primary tumor growth and metastasis of the 4T1-1V line are shown in Figure 3 and Table 2. Biophotonic imaging of animals each week over a six week period after implantation of 4T1-1V cells in the abdominal no. 9 (or no. 4) mammary fat pad revealed several previously unidentified characteristics of the 4T1 model. Tumor growth at the site as measured by biophotonic imaging was found to occur in a biphasic fashion with rapid growth during the first two weeks, regression between weeks 2 and 4, and increased growth again in weeks 5 and 6 (Fig. 3, Top Panels). Metastasis became apparent in the thoracic region and lower limbs in weeks 5 and 6 of the second growth phase although metastasizing cells probably seeded these sites earlier [8,9]. Examination of light emission from organs removed from the animals at week 6, revealed a spectrum of organ metastasis similar to that observed for the 4T1-12B line.

**Table 2:** Kinetics and extent of metastasis for 4T1-1V^1^

**Time after injection (days)**	**Primary**	**Lungs**	**Spleen**	**Liver**	**Bone**	**Kidney/Adrenals**	**Other**
8–14	3(3)^2^	-	-	-	-	-	-
15–21	3(3)	1(3)	-	-	-	-	-
22–28	5(5)	5(5)	1(5)	2(5)	0(5)	1(5)	Intestine 1(5)
29–35	7(7)	7(7)	2(7)	6(7)	3(7)	2(7)	Intestine 1(7)
36–42	7(7)	7(7)	5(7)	6(7)	6(7)	2(7)	Brain 1(7) Heart 1(7)

^1^Cells were introduced into fatpads of normal female BALB/c mice and imaged at the indicated times as described in MATERIALS AND METHODS.

^2^Number of animals positive (total number of animals)

**Figure 4:**
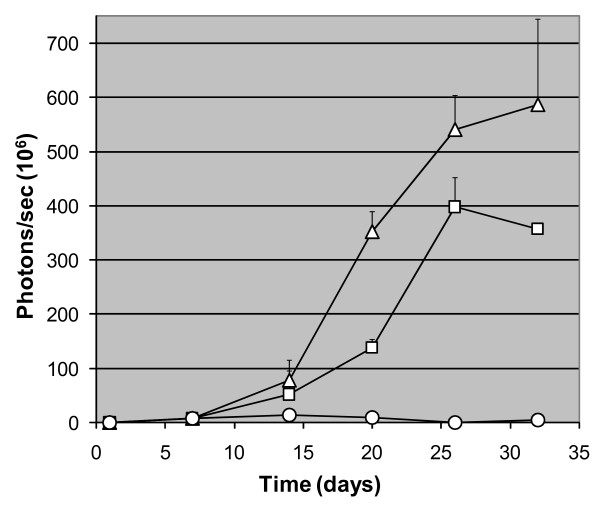
**Involvement of immune system in primary tumor growth**. 4T1-12B cells (10^6^) were implanted into the mammary fat pad of two normal (○), athymic nude (□) and SCID (△) BALB/c mice and imaged weekly as described in MATERIALS AND METHODS. Average luminescence +/- sd for each time point is plotted.

Further analysis revealed that biphasic growth at the primary site was related to immune system function. The regression that was observed in weeks 2 through 4 in normal BALB/c mice was associated with necrosis and infiltration of leukocytes (Fig. 3, Bottom Right Panel). Biphasic tumor growth did not occur in athymic nude or SCID BALB/c mice (Fig. 4) suggesting involvement of an acquired immune response in the effect. Antibodies directed against multiple 4T1 cell antigens were found in the sera of mice at week 6 (data not shown) further supporting involvement of an acquired immune system response in the regressive process.

Imaging of animals and organs at various times after introduction of 4T1-1V cells in the fat pad revealed a clear progression of metastasis first to lungs (beginning around 3 weeks) and later to liver, bone and spleen (weeks 3–6) with occasional metastasis to brain, heart and intestines at the later times (Table 2). Tumor cells were detected in lymph nodes adjacent to primary tumors and elsewhere in the animal consistent with previous studies suggesting that 4T1 cells metastasize via the lymphatic system as well as hematogenously [9,12].

**Figure 5:**
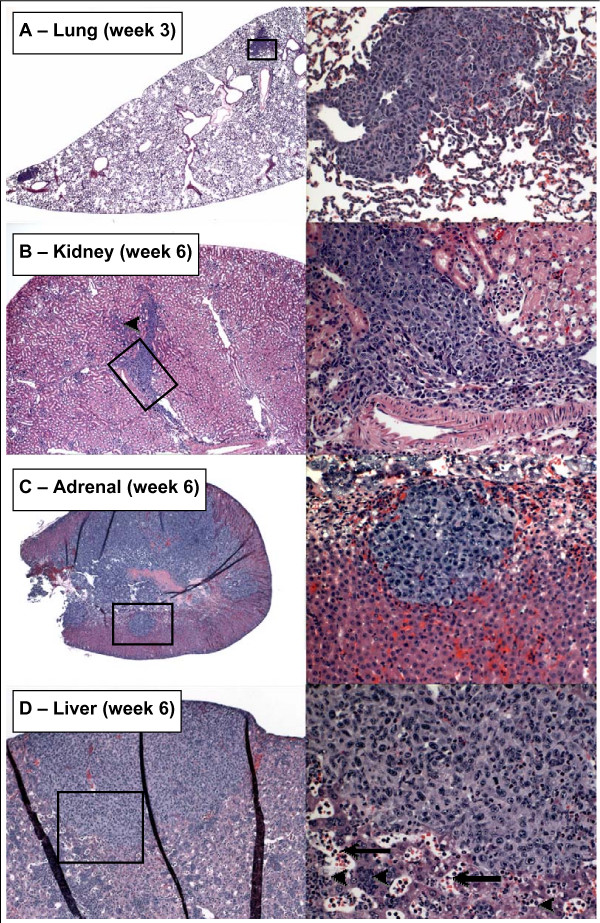
**Metastasis to lungs, kidneys, adrenals and liver**. (**A**) Lung at 3 weeks showing metastases adjacent to blood vessels. (**B**) Tumor cells in a major vessel of the kidney at week 6. Note infiltration of tumor cells into kidney parenchyma (arrowhead, left panel). (**C**) Tumor-laden adrenal gland at 6 weeks with multiple spherically-shaped metastases. (**D**) Large metastasis on the surface of the liver at week 6. Note abnormal appearance of liver parenchyma and high levels of leukocytes in parenchyma (arrowheads, right panel) and sinusoids (arrows, right panel). Specimens were obtained from the experiment described in Figure 3.

**Figure 6:**
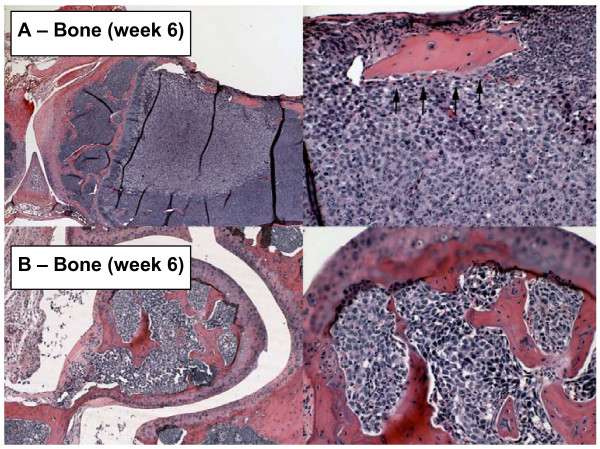
**Metastasis to bone**. **(Top Left) **Metastasis near joint between femur and tibia at week 6. Note extensive degradation of bone adjacent to the upper surface of the tumor. (**Top Right**) Interface between tumor and bone at higher magnification. Note osteoclasts (arrowheads) lining the lower surface of the bone. (**Bottom Left and Right**) Femoral metastasis within the joint itself at low and high resolution at 6 weeks. Specimens were obtained from the experiment described in Figure 3.

The results of histological examination of metastases at selected times and sites are shown in Figure 5. In lungs and kidneys metastases were found within or in close proximity to afferent vessels and in most cases appeared infiltrative. In adrenals and liver metastases were more localized, often appearing spherical in nature. Metastasis to bone was prevalent throughout the skeletal system including skull, ribs, sternum, and limbs. Bone-associated osteoclasts were often observed in areas adjacent to bone metastases indicating increased osteoclastogenesis and elevated degradation of bone in these areas (Fig. 6).

**Figure 7:**
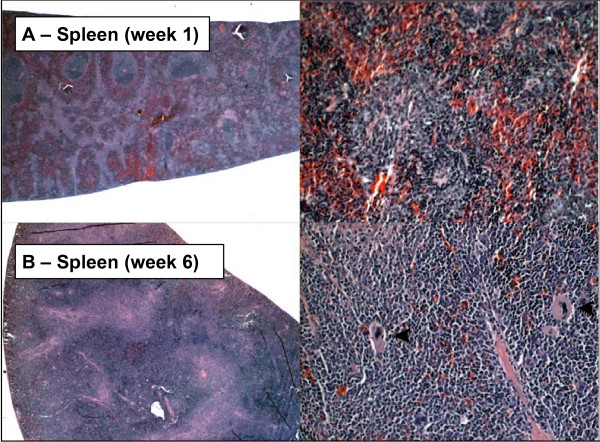
**Hematopoiesis in spleen**. Spleen at 1 (**Top**) and 6 (**Bottom**) weeks. Spleen appears normal at week 1. Extensive extramedullary hematopoiesis is apparent at week 6 as evidenced by the presence of megakaryocytes (arrow heads). Specimens were obtained from the experiment described in Figure 3.

**Figure 8:**
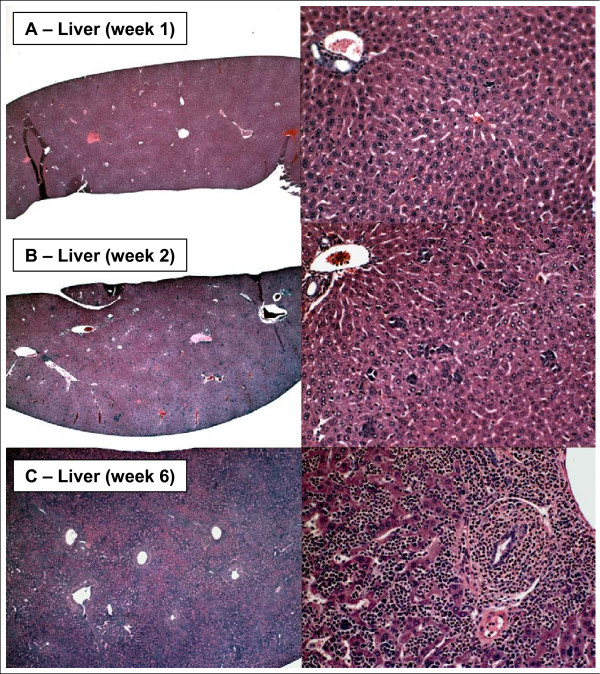
**Hematopoiesis in liver**. Liver at 1 (**Top**), 2 (**Middle**) and 6 (**Bottom**) weeks. Liver appears normal at week 1. Islands of extramedullary hematopoiesis are seen at week 2 and extensive hematopoiesis throughout the liver is apparent at week 6. Note increased proportion of nucleated cells in blood vessels at weeks 2 and 6. Specimens were obtained from the experiment described in Figure 3.

A progressive increase in hematopoiesis was observed throughout the 6 week time course as primary tumors progressed and metastases developed at distant sites. This was evidenced by increasing levels of circulating neutrophils and other leukocytes (Table 3) and by enlargement of the spleen and liver resulting from extramedullary hematopoiesis that developed in these organs (Figs. 7 and 8). Extramedullary hematopoiesis was apparent by week 2 when primary tumors began to regress and continued to increase until death ensued between weeks 6 and 8. Immature myelocytic cells (Band N) were found in the circulation at week 4. The histology of spleen and liver and the composition and levels of circulating leukocytes are consistent with expansion of granulocyte lineages with circulating leukocytes reaching leukemia-like levels by the end of the observation period (6 weeks).

**Table 3:** Circulating white cell analysis^1^

**WBC Population**	**Control (n = 1)**	**Week 1 (n = 3)**	**Week 4 (n = 4)**
WBC	5.300^2^	4.200	56.210
		4.200	66.000
		2.800	15.700
			134.000
Seg N	0.424 (8%)^3^	1.386 (33%)	34.850 (62%)
		0.546 (13%)	48.840 (74%)
		1.148 (41%)	8.164 (52%)
			99.160 (74%)
Band N	-	-	3.373 (6%)
		-	3.962 (6%)
		-	0.157 (1%)
			10.720 (8%)
Metamyelocytes	-	-	0.562 (1%)
		-	0.660 (1%)
		-	-
			2.680 (2%)
Lymphocytes	4.823 (91%)	2.772 (66%)	16.863 (30%)
		3.654 (87%)	10.560 (16%)
		1.624 (58%)	6.594 (42%)
			20.100 (15%)
Monocytes	-	-	-
		-	1.320 (2%)
		-	0.314 (2%)
			-
Eosinophils	0.053 (1%)	0.042 (1%)	0.562 (1%)
		-	0.660 (1%)
		0.028 (1%)	0.471 (3%)
			1.340 (1%)

^1^Whole blood was collected by cardiac puncture at the indicated time after orthotopic introduction of tumor cells as described in MATERIALS AND METHODS.

^2^Total number of WBC/ml blood in millions for each animal tested.

^3^Number of cells/ml blood in millions for each animal tested (% of total WBC for animal)

### Genes associated with the 4T1 metastatic phenotype

**Figure 9:**
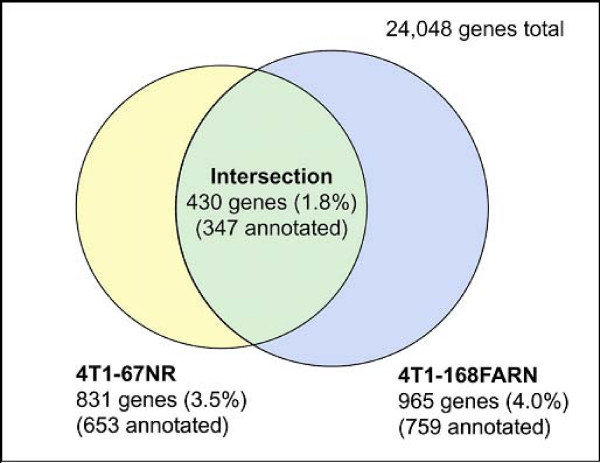
**Genes with altered expression in 4T1 vs. 67NR and 168FARN**. Genes with 2-fold expression differences for 4T1 vs. 67NR and 4T1 vs. 168FARN were determined as described in MATERIALS AND METHODS. Genes in the intersection between the two comparisons are those considered to be associated the metastatic phenotype of the 4T1 cell line. Genes in the intersection represent 1.8% of the total genes analyzed, 52% of genes differentially expressed for 4T1 vs. 67NR and 45% of those differentially expressed for 4T1 vs. 168FARN.

Gene expression analysis was carried out on the 4T1 cell line and two of its sibling lines, 67NR and 168FARN to identify expression differences associated with the 4T1 metastatic phenotype. Both of the sibling lines are non-metastatic when introduced orthotopically into BALB/c mice [9]. The 67NR line displays little if any dissemination from the primary site, whereas the 168FARN line displays dissemination to lymph nodes, but not to blood or distant organs [9]. Using Illumina MouseRef-8 BeadChip arrays, multiple replicates, and carefully controlled culture conditions, highly significant (p < 0.0001) expression data for differences as low as 1.2-fold were achieved. Of the 24,048 genes represented on the arrays, 1.8% or 430 genes (347 annotated) differed by 2-fold or more in the 4T1 line relative to the other two lines (Fig. 9). The median false discovery rate for these genes was less than 1 in 500. The majority of all 2-fold differences were found to be significant (p > 0.0001) for both the 4T1/67NR (66.4%) and 4T1/168FARN (98.7%) comparisons. These results indicate a very high level of confidence in the genelists that were produced from the data.

Ingenuity Pathway Analysis (IPA) of genes differentially expressed in 4T1 relative to the two non-metastatic lines revealed significant association with cancer and other diseases including hematological and inflammatory disease (Table 4), findings consistent with the high level of inflammation and hematopoiesis observed for the 4T1 lines *in vivo*. Also consistent with the 4T1 metastatic phenotype was association with cell movement, cell signaling, cell growth, proliferation and death, and cell to cell signaling and interaction (Table 4). Many of the expression differences that characterize the 4T1 phenotype including those known to be involved in metastasis and/or tumorigenesis are listed in Additional File 1.

**Table 4:** Ontological analysis^1^

	**p-value**	**# molecules**
**Diseases and Disorders**		

Cancer	5.07E-14-5.72E-04	100
Hematological Disease	1.80E-08-5.72E-04	51
Connective Tissue Disorders	3.41E-08-1.44E-04	35
Dermatological Diseases and Conditions	1.75E-07-5.37E-04	41
Inflammatory Disease	8.81E-07-5.51E-04	38

**Molecular and Cellular Functions**		

Cell Movement	1.18E-15-5.72E-04	69
Cell Signaling	1.30E-13-2.04E-04	106
Cell Death	1.67E-13-5.32E-04	96
Cellular Growth and Proliferation	1.03E-12-5.79E-04	111
Cell to Cell Signaling and Interaction	1.94E-08-5.19E-04	63

^1^304 annotated gene were included in the analysis

**Figure 10:**
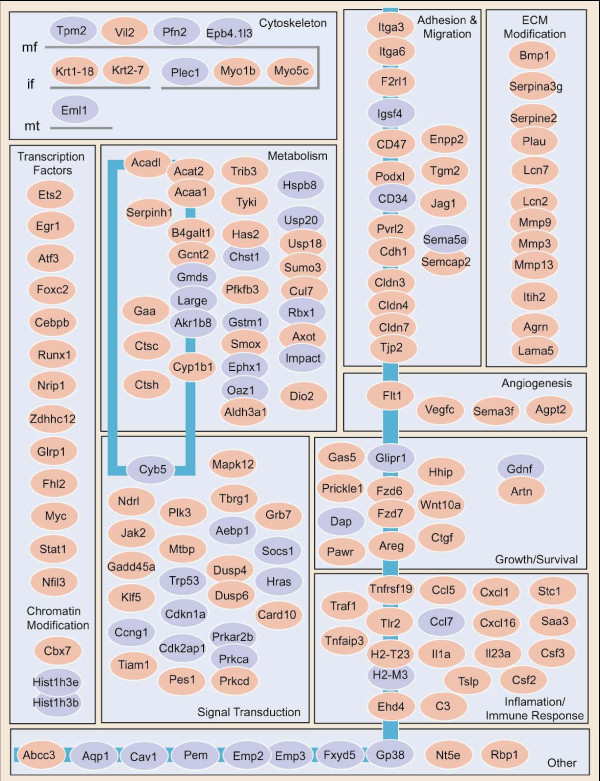
**4T1 genes categorized by cellular location and function**. Genes associated with the 4T1 metastatic phenotype that fall into the categories shown are listed in the figure. Genes shown in red are elevated in 4T1 and genes shown in blue reduced in 4T1. The blue line to the right of middle represents the plasma membrane with genes falling to the right of it representing secreted genes. The blue rectangle to the left of middle represents intracellular membranes and genes falling inside the rectangle are genes located within intracellular organelles.

Genes differentially expressed in 4T1 were categorized with respect to cellular location and function (Fig. 10). Among the genes are substantial numbers involved in cell adhesion, migration, angiogenesis, and extracellular matrix modification; cytoskeleton function; cell proliferation, apoptosis and survival; cellular metabolism; and inflammation and immune response. Altered expression of several transcription factors and genes involved in chromatin modification that regulate these processes were also observed. Elevated expression of genes associated with tight junctions (Cldn3, Cldn4, Cldn7 and Tjp2), adherins junctions (Cdh1 and Vil1) focal adhesions (Itga3, Itga6 and Lama5), and intermediate filaments (Krt1-18 and Krt2-7) indicate that the 4T1 line has greater epithelial character than the non-metastatic lines. An increased propensity for extracellular matrix (ECM) remodeling is suggested by elevated expression of matrix metalloproteinases (Mmp3, Mmp9 and Mmp13), urokinase-type plasminogen activator (Plau) and secreted protease inhibitors (Serpina3g, Serpin2 and Lcn2).

#### Signaling pathways associated with phenotype

**Figure 11:**
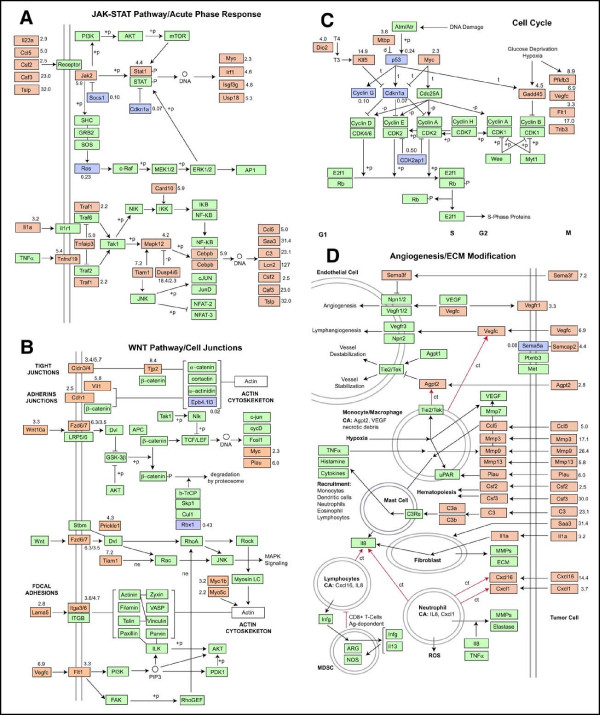
**Pathways involved in metastatic phenotype of 4T1**. Pathways shown are based on known Kegg pathways with modifications based on recent literature relating 4T1 phenotype genes to these pathways. Red and blue boxes represent genes that are up-regulated or down-regulated by > 2-fold, respectively. Red arrows represent chemotaxis. Numbers outside the boxes give the higher of the two ratios for expression in 4T1 relative to the non-metastatic 67NR and 168FARN lines.

Several signaling pathways appear to be activated in 4T1 cells (Fig. 11). Most conspicuous is activation of the Jak/Stat pathway as indicated by elevated expression of Jak2 and Stat1, decreased expression of Socs1 and increased expression of several Stat target genes (Myc, Irf1, Igsf3g and Usp20) (Fig. 11A). Also conspicuous is activation of p38 MAPK (Mapk12) as indicated by increased expression of CCAAT/enhancer binding protein beta (Cebpb) and high levels of expression of Cebpb/NFκB target cytokines (Ccl5/RANTES, Csf2, Csf3 and Tslp) and acute phase proteins (Saa3, C3 and Lcn2). Increased expression of TIAM1 (Tiam1) and genes in the IL-1 and TNF-α pathways (Il1a, Tnfrsf19, Traf1, Card10) suggest that these pathways, which are known to activate p38 MAPK, may be involved in the expression of Cebpb/NFκB targets. Targets of p38 MAPK are known to activate the Jak/Stat pathway (Fig. 11) so it is therefore likely that p38 MAPK signaling is responsible for the activation of the Jak/STAT pathway in these cells. Elevated expression of Wnt (Wnt10a) and its receptors (Fzd6 and Fzd7) suggests activation of the Wnt pathway (Fig. 11B). While some Wnt pathway targets (Myc and Plau) displayed elevated expression, other known targets (c-jun, cycD and Fosl1) did not. It may be that the purpose of altered expression of Wnt pathway ligand and receptors is to increase β-catenin levels to support junctional complexes that are more prevalent in these cells. Both the canonical and the non-canonical Wnt pathway are known to play an important role in establishment and maintenance of cellular junctions [16]. Finally, 4T1 displayed significantly reduced levels of CDK2-associated protein 1 (CDK2ap1), p53 and two p53 targets, cyclin-dependent kinase inhibitor p21 (Cdkn1a) and cyclin G (Ccng1) (Fig. 11C). These alterations would be expected to accelerate the early phase of the cell cycle (G_1 _→ S) and attenuate the DNA damage response. Expression of a third p53 target, Gadd45, was elevated. Gadd45 is regulated by hypoxia and glucose deprivation as well as by p53. Elevated expression of several genes known to be sensitive to hypoxia and/or glucose deprivation (Pfkfb3, Vegfc, Flt1 and Trib3), suggest that elevated expression of Gadd45 may be due to these factors and that 4T1 cells exist in a state of stress or pseudo-stress even under optimal culture conditions.

#### Alterations related to tumor microenvironment

A variety of factors produced at elevated levels by 4T1 cells are secreted cytokines, chemokines, acute phase proteins and proteases that interact locally and systemically with the host to produce, recruit and activate cells of hematopoietic origin capable of remodeling the tumor microenvironment and facilitating tumor cell dissemination. These factors and their expected effects on the tumor microenvironment are depicted in Figure 11D. Two important modulators of endothelial cell function produced by 4T1 cells are vascular endothelial growth factor C (Vegfc) and angiopoietin 2 (Agpt2). VEGF-C interacts with VEGF receptors on endothelial cells to stimulate angiogenesis and lymphangiogenesis when existing vessels are destabilized. Angiopoietin 2 destabilizes vessels by antagonizing the stabilizing effects of angiopoietin 1. Together, these factors would be expected to induce both angiogenesis and lymphangiogenesis, increase tumor vascularization and provide routes of escape of tumor cells. The inhibitory effect of semaphorin 3F (Sema3f) on angiogenesis would be expected to shift vessel development toward lymphangiogenesis. Angiopoietin 2 and VEGF-C also serves as chemotactic factors for recruitment of circulating monocytes and macrophages.

Macrophages and other cells recruited to the tumor are produced in the bone marrow and other tissues by hematopoiesis. Colony stimulating factors GM-CSF (Csf2) and G-CSF (Csf3) produced and secreted by 4T1 cells stimulate hematopoiesis along the myeloid lineages and are likely to be responsible for the high levels of hematopoiesis and circulating leukocytes observed when tumors from 4T1 cells are established *in vivo *[17]. Several factors produced by 4T1 cells are known to play a role in recruitment of hematopoietic cells to tissues. RANTES (Ccl5) is chemotactic for mast cells [18] and fragments generated autocatalytically from complement C3 (C3) are capable of stimulating mast cells to release TNFα, histamine, cytokines and other factors that can act to recruit a wide range of cells including monocytes, dendritic cells, neutrophils, eosinophils and lymphocytes. RANTES (Ccl5) is also known to stimulate secretion of interleukin 8 (Il8) from macrophages [19]. Interleukin 8 is also released by stromal fibroblasts in response to interleukin 1α produced by 4T1 cells. Interleukin 8 along with chemokines (Cxcl6, Cxcl1) released by 4T1 are chemotactic for neutrophils [20,21]. Finally, matrix metalloproteinases produced by macrophages, fibroblasts and neutrophils recruited to the tumor would add to the already high levels of matrix metalloproteinases released by the tumor cells themselves thereby creating a high potential for dissolution of matrix and cell-matrix interactions, a condition likely to facilitate tumor cell invasion and metastasis.

Previous studies have indicated that populations of immature myeloid cells called myeloid derived suppressor cells (MDSC) are induced by tumors and that these cells facilitate tumor growth and metastasis by suppressing the immune response [22]. Ectopic expression of interleukin 1β in 4T1 cells has been shown to increase MDSC levels and stimulate growth and metastasis of 4T1 tumors *in vivo *[23]. Because interleukin 1α rather than interleukin 1β is the predominant form of interleukin 1 produced by 4T1 cells, and because the two cytokines have similar biological activity, it is likely that expression of interleukin 1α by 4T1 is involved in production of MDSC and their effects on growth and metastasis of 4T1 *in vivo*.

## Discussion

Here we report on the generation of two clonal 4T1 cell lines (4T1-12B and 4T1-1V), both of which stably express firefly luciferase at a high level in the absence of selective pressure, and one (4T1-1V) which was also modified by addition of an FRT site in its genome. These lines were shown to have metastatic characteristics similar to the parental 4T1 line displaying metastasis to bone, lungs, and liver and brain organs primarily affected in human breast cancer. The ability to image the cells *ex vivo *with high sensitivity allowed detection and quantitation of metastases in affected organs more effectively than has been possible previously. Luciferase-expressing 4T1 variant cell pools and lines with increased propensity for metastasis to brain, liver, and bone have recently been isolated (to be published elsewhere). These variants will further expand the repertoire of syngeneic models available for the study of late stage breast cancer.

An acquired immune response was found to play an important role in regulating 4T1 tumor growth and metastasis. 4T1 tumors established in normal BALB/c mice displayed a substantial loss of tumor cells beginning 2–3 weeks after introduction. This effect was not apparent in BALB/c nude and BALB/c SCID mice, in which 4T1 cells in tumors proliferated rapidly and continuously. Antibodies directed against several 4T1 antigens were detected in sera from normal tumor-bearing BALB/c mice further supporting the involvement of an acquired immune response to the cells. Myeloid derived suppressor cells (MDSC) which are known to be induced in 4T1 tumor-bearing mice are likely to be involved in establishment and maintenance of 4T1 tumors by attenuating the immune response to allow survival of the tumor in weeks 3–4 and re-emergence of tumor growth in weeks 5–6. Further work will be required to determine the actual role that MDSC and other immune system components play in regulating the growth and survival of 4T1 tumors.

Metastasis of 4T1 tumors is associated with extensive necrosis and inflammation within the primary tumor and hematopoiesis in several mouse organs including spleen and liver. Elevated hematopoiesis has recently been reported for the 4T1 model [17,24]. Whether or not a causal relationship exists between these processes and metastasis remains to be demonstrated although two observations suggest that there may be such a connection. First, the extent of necrosis is greater in 4T1 tumors than those derived from less metastatic sibling cell lines (67NR, 168FARN) as indicated by the occurrence of large areas of visible necrosis in the 4T1 tumors. Second, a causal relationship between inflammation and metastasis is supported by the inhibitory effect of the COX-2 inhibitor, SC-236, on metastasis of 4T1 after primary tumor excision [25]. Inflammation is known to have a positive effect on metastasis in several systems [26-28] and is likely to have a pro-metastatic effect in this system as well.

Inflammation in metastatic tumors is generally thought to result from signals produced by dying cells and ECM fragments in areas of insufficient vascularization [29]. A noteworthy finding of this study is that 4T1 tumor cells, when cultured under optimal growth conditions, produce a wide range of factors capable of inducing production, recruitment and activation of inflammatory cells. These factors include colony stimulating factors GM-CSF (Csf2) and G-CSF (Csf3); cytokines Ccl5, Cxcl1, Cxcl6 and Tslp; angiogenic factors Agpt2 and Vegfc; and acute phase proteins Saa3, C3, and Lcn2. While this does not preclude the involvement of cell death in initiating an inflammatory response in the tumor, it does suggest that the tumor cells themselves may play a more direct and active role in directing pro-metastatic inflammatory processes than previously envisioned.

The methodology used in this study for analysis of gene expression yielded highly significant data characterizing the 4T1 metastatic phenotype. The majority of genes that differed by more than 2-fold in 4T1 relative to the two non-metastatic sibling lines examined displayed an exceptionally high level of significance (p < 0.0001) and genelists obtained at this level of significance displayed very low false positive rates. The statistics argue that the results obtained provide a relatively complete and accurate picture of expression differences associated with the 4T1 phenotype. The high level of accuracy and reproducibility that was achieved is attributed to use of the Illumina BeadChip platform and analysis of cells cultured under carefully controlled growth conditions that minimize differences between biological replicates. The data obtained from this study provide detailed information regarding the genes and pathways involved in breast cancer progression for this model and will be particularly useful for further analysis of the pathological processes responsible for progression to a metastatic phenotype.

Unlike many cell lines used as xenograft models, subclones of the 4T1-12B cell line that had undergone more than 20 doublings were found to be homogeneous with respect to metastatic properties. These cells also display a high plating efficiency and no visibly apparent differentiation in culture or *in vivo*. Thus, the cells resemble stem cells found in populations of cell lines such as MCF7 [30] in that they are self renewing, but differ in that they do not appear to differentiate. While more work is need to determine the basis for this property, the characteristic has utility for studies aimed at determining gene function since clonal lines in which a specific genes have been over-expressed or knocked down can be expected to retain the properties of the parental line from which they were derived. In this regard, the FRT site in the 4T1-1V line will be useful for production of isogenic lines for analysis of gene function.

## Conclusion

In conclusion, this study provides basic information for those interested in using two imagable 4T1 breast cancer models developed in this laboratory. Several characteristics of these models make them particularly attractive for the study of late stage breast cancer. First and foremost, because of their syngeneic nature, they provide a highly physiologic system suitable for analysis of innate and acquired immune system roles in tumor growth and metastasis. The relatively complete gene expression data provided offer numerous avenues for further study of the molecular and pathologic basis for these and other processes related to late stage breast cancer. To our knowledge, the 4T1 model is the only system that has the capacity to metastasize to all organs affected in breast cancer in humans when introduced orthotopically. For this reason, and because of the ease of use and reproducibility that can be achieved, these imagable models provide ideal systems for determining anti-metastatic effects of cancer drugs and therapeutic regimens and is well suited for investigating the molecular, cellular and pathologic basis for metastasis to specific organs and tissues.

## Abbreviations

cRNA: complementary RNA; DMEM: Dulbecco's Minimum Essential Medium; ECM: extracellular matrix; FBS: fetal bovine serum; FLP: flippase; FRT site: FLP recombinase targeting site; GEM: genetically engineered mouse; IPA: Ingenuity Pathway Analysis; MDSC: myeloid derived suppressor cells; NCS: normal calf serum; NEAA: nonessential amino acids; PBS: phosphate buffered saline; Q-PCR: quantitative PCR; ROI: region of interest; siRNA: small inhibitory RNA; shRNA: short hairpin RNA.

## Competing interests

Cell lines described in this study are licensed by Tufts University for commercial use. Royalties are split between Tufts University (including GGS), Wayne State University and the NIH.

## Authors' contributions

KT acquired and analyzed imaging data. MF acquired and analyzed imaging and gene expression data. JA evaluated histology data. GGS conceived of the study, analyzed imaging data, and wrote the manuscript. KT and MF contributed equally to the study. All authors read and approved the final manuscript.

## Pre-publication history

The pre-publication history for this paper can be accessed here:



## Supplementary Material

Additional File 1 Gene table. A list of genes associated with the 4T1 metastatic phenotype. Click here for file
